# Nuclear mRNPs are compact particles packaged with a network of proteins promoting RNA–RNA interactions

**DOI:** 10.1101/gad.350630.123

**Published:** 2023-06-01

**Authors:** Fabien Bonneau, Jérôme Basquin, Barbara Steigenberger, Tillman Schäfer, Ingmar B. Schäfer, Elena Conti

**Affiliations:** 1 Department of Structural Cell Biology, Max Planck Institute of Biochemistry, Martinsried/Munich D-82152, Germany;; 2 Mass Spectrometry Core Facility, Max Planck Institute of Biochemistry, Martinsried/Munich D-82152, Germany;; 3 Electron Microscopy Core Facility, Max Planck Institute of Biochemistry, Martinsried/Munich D-82152, Germany

**Keywords:** THO, TREX, Sub2, UAP56, EJC, CHTOP, ERH, IDR, ribonucleoprotein particles, cryo-electron tomography (cryo-ET), cross-linking mass spectrometry (XL-MS), RNA annealing

## Abstract

Messenger RNAs (mRNAs) are at the center of the central dogma of molecular biology. In eukaryotic cells, these long ribonucleic acid polymers do not exist as naked transcripts; rather, they associate with mRNA-binding proteins to form messenger ribonucleoprotein (mRNP) complexes. Recently, global proteomic and transcriptomic studies have provided comprehensive inventories of mRNP components. However, knowledge of the molecular features of distinct mRNP populations has remained elusive. We purified endogenous nuclear mRNPs from *Saccharomyces cerevisiae* by harnessing the mRNP biogenesis factors THO and Sub2 in biochemical procedures optimized to preserve the integrity of these transient ribonucleoprotein assemblies. We found that these mRNPs are compact particles that contain multiple copies of Yra1, an essential protein with RNA-annealing properties. To investigate their molecular and architectural organization, we used a combination of proteomics, RNA sequencing, cryo-electron microscopy, cross-linking mass spectrometry, structural models, and biochemical assays. Our findings indicate that yeast nuclear mRNPs are packaged around an intricate network of interconnected proteins capable of promoting RNA–RNA interactions via their positively charged intrinsically disordered regions. The evolutionary conservation of the major mRNA-packaging factor (yeast Yra1 and Aly/REF in metazoans) points toward a general paradigm governing nuclear mRNP packaging.

## Abstract (precis)

In this study, Bonneau et al. describe an efficient strategy to isolate and access the molecular and structural properties of mRNA ribonucleoprotein (mRNP) complexes. They show that mRNPs exist in compact particles and that mRNPs with positively charged intrinsically disordered domains facilitate RNA–RNA interactions in yeast and human cells.

The synthesis of eukaryotic mRNAs is coupled to cotranscriptional processing events, such as 5′ capping, splicing, and 3′ poly(A) tail addition (Müller-McNicoll and Neugebauer 2013). These biogenesis steps not only modify the ribonucleotide chains, but also coat the transcripts with proteins (Singh et al. 2015). If all biogenesis steps occur correctly, the resulting mature mRNPs are transported through nuclear pore complexes by export factors. Failure in essentially any step of the biogenesis process can result in malformed mRNPs that are retained in the nucleus and eliminated by nuclear quality control pathways (Garland and Jensen 2020). A well-known example is the exosome-mediated degradation of aberrant mRNAs generated in yeast strains harboring deletions or mutations of the mRNP biogenesis factors *hpr1*, *sub2*, or *yra1* (Libri et al. 2002; Rougemaille et al. 2007). Hpr1 is a subunit of THO, a multiprotein complex that was originally identified for its cotranscriptional role in preventing nascent mRNA–DNA hybrids, the so-called R-loops (Luna et al. 2020). Sub2 is a DEAD-box RNA-dependent ATPase that can interact with THO (Pühringer et al. 2020; Schuller et al. 2020; Xie et al. 2021) and with Yra1 (Jensen et al. 2001; Sträßer and Hurt 2001). Yra1 is an essential hnRNP-like protein that was originally discovered in yeast as a factor with potent RNA-annealing activity (Portman et al. 1997; Infantino and Stutz 2020).

THO, Sub2 (UAP56), and Yra1 (Aly/REF) are evolutionarily conserved from lower to higher eukaryotes (Stutz et al. 2000). In yeast, THO recruits Sub2 and Yra1 upon transcription elongation, a recruitment process required for the formation and export of stable mRNPs (Lei et al. 2001; Zenklusen et al. 2002; Abruzzi et al. 2004). In human cells, Aly/REF is recruited to intron-containing mRNAs by the splicing process (Masuda et al. 2005; Viphakone et al. 2019). However, Aly/REF proteins also associate with intronless mRNAs (Taniguchi and Ohno 2008). For both types of transcripts, Aly/REF deposition requires the ATPase activity of UAP56 (Taniguchi and Ohno 2008; Dufu et al. 2010). Mutations or deletion affecting THO, Sub2, and Yra1 (or their human orthologs) result in similar phenotypes; namely, transcription defects (hyperrecombination and R-loop stabilization) and export defects, which can be collectively attributed to a failure in cotranscriptional mRNP assembly (Infantino and Stutz 2020; Luna et al. 2020). Notwithstanding the commonly accepted existence of a defined THO–Sub2–Yra1 transcription export (TREX) protein–protein complex (Sträßer et al. 2002), the molecular mechanisms with which THO, Sub2, and Yra1 contribute to the formation of mature mRNPs remain unclear.

A related, and perhaps bigger, unknown pertains to the makeup of mRNPs. Global proteomic studies have identified hundreds of mRNA-binding proteins (Hentze et al. 2018). However, knowledge of the complement of proteins of distinct mRNP populations (for example, in a given cellular compartment or at a given stage of their life cycle) is still superficial. While in the past two decades there has been tremendous progress in the characterization of large and dynamic ribonucleoprotein complexes such as ribosomes and spliceosomes, the sheer diversity, complexity, and transient nature of mRNPs has posed a great challenge to their isolation and structural characterization. In this context, it is perhaps not surprising that to date there is little information on mRNP architecture, particularly at a level conducive to a mechanistic understanding. Negative stain electron microscopy (EM) studies of complexes isolated from *Saccharomyces cerevisiae* (Batisse et al. 2009) and in situ EM tomography studies of Balbiani ring particles from the dipteran *Chironomus tentans* (Björk and Wieslander 2015) have shown the presence of defined particles. Together with recent single-molecule fluorescence in situ hybridization (Adivarahan et al. 2018; Khong and Parker 2020) and proximity ligation studies (Metkar et al. 2018) on mammalian mRNPs, the emerging picture is that pretranslational mRNPs are dense particles in which the mRNA is compacted. While this condensation is conceptually reminiscent of DNA compaction in chromatin, there is no known nucleosome equivalent acting as a generic mRNA structural organizer. In this work, we pushed the leading edge of mRNP biochemistry combined with integrative structural approaches (such as cryo-electron tomography, cross-linking mass spectrometry, and AlphaFold predictions) to allow molecular-level investigations of a nuclear mRNP population using budding yeast as a model system.

## Results

### Isolation of endogenous yeast THO–Sub2-containing assemblies

We set out to isolate nuclear mRNPs from *S. cerevisiae*. For affinity capture of nuclear mRNPs from their native environment, we used a variation of the original tandem affinity purification (TAP) method (Rigaut et al. 1999). After engineering several yeast strains to identify an endogenous tagging system that would not abrogate cellular fitness and would result in effective mRNP purification, we honed in on protein A (ProtA) and Twin-Strep (TS) as affinity tags and on Sub2 and the THO complex component Hpr1 as bait. Inspired by previous interactome studies (Oeffinger et al. 2007; LaCava et al. 2016) and expecting mRNPs to be large, transient, and RNase-sensitive assemblies, we minimized the time needed for the isolation process (to limit the action of RNases and preserve the integrity of the particles without resorting to cross-linking) and the affinity matrix (to avoid size selection effects unsuitable for large macromolecular assemblies).

**Figure 1.**
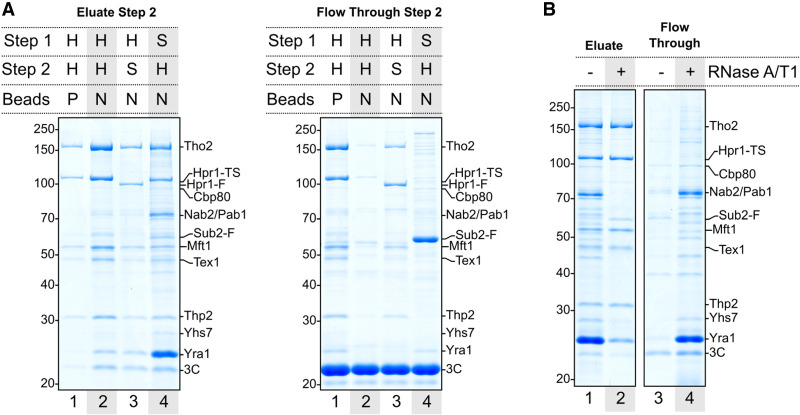
Isolation of *S. cerevisiae* nuclear mRNPs. (*A*) Coomassie-stained 10% SDS-PAGE gels of SDS eluates (*left*) or TCA-precipitated unbound fractions (*right*) from the final purification step. The first affinity capture (step 1, via protein A) was always performed using nonporous magnetic beads. Elution in the first step was carried out with 3C protease. The second affinity capture (step 2, Twin-Strep) was performed using either porous (P) or nonporous (N) magnetic beads. H (Hpr1) and S (Sub2) indicate the tagged protein used as bait in the corresponding affinity capture step. Band annotations are based on mass spectrometry fingerprinting (Supplemental Fig. S1) and include the presence of tags remaining after elution. (F) FLAG, (TS) Twin-Strep. (*B*) Coomassie-stained 10% SDS-PAGE gels of SDS eluates (*left*) or TCA-precipitated unbound fractions (*right*) with or without RNase A/T1 mix treatment on beads prior to elution from the second affinity step.

Using nonporous magnetic beads for both steps of the purification process was crucial to allow retention of large-sized complexes, as exemplified by affinity captures from a yeast strain where a single mRNA biogenesis factor (Hpr1) was fused C-terminally with both ProtA and TS tags in tandem (the *hpr1-TS-3C-ProtA* yeast strain) (Fig. 1A). After performing the initial affinity capture step with antibody-conjugated magnetic beads for ProtA purification, we eluted the complexes by 3C protease cleavage and subjected them to a second purification step via the TS epitope, eluting the final samples with SDS-PAGE buffer for visual inspection on Coomassie-stained gels. When a standard Strep-Tactin sepharose matrix was used in the second enrichment step, we isolated Hpr1 together with the other THO complex subunits (Fig. 1A, left panel, lane 1). However, the eluted fraction contained fairly low amounts as compared with the flowthrough fraction (Fig. 1A, right panel, lane 1). Since the size selection properties of porous matrices are unfavorable for large complexes (Oeffinger et al. 2007), we purposefully made nonporous Strep-Tactin beads. Using nonporous beads also for the second enrichment step significantly improved the yield (Fig. 1A, left panel, cf. lanes 1 and 2), with little material in the flowthrough (Fig. 1A, cf. lanes 2). The eluted sample showed not only the presence of all THO complex subunits, but also the appearance of additional protein bands, albeit clearly substoichiometric with respect to THO (Fig. 1A, left panel, lane 2).

Reasoning that the substoichiometry of the additional protein bands may reflect the presence of a heterogeneous mixture of different Hpr1-containing complexes in the single-bait affinity capture, we used a bimolecular affinity purification strategy, fusing the ProtA and TS tags on either Hpr1 or Sub2. Carrying out the first ProtA affinity capture step via Hpr1 and then enriching for Hpr1-containing complexes containing Sub2 (from a *hpr1-FLAG-3C-ProtA/Sub2-TS* yeast strain) resulted in a fairly low yield of THO-containing complexes (Fig. 1A, left panel, lane 3), possibly because not all THO-containing complexes may contain Sub2 or because the TS tag directly fused to the C terminus of Sub2 may not be sufficiently accessible. When performing the first ProtA affinity capture step via Sub2 and then enriching for Sub2-containing complexes containing Hpr1 (from a *hpr1-TS/Sub2-FLAG-3C-ProtA* yeast strain), the eluted fraction showed a high yield of THO-containing complexes, while very little material remained in the flowthrough (except for a portion of Sub2 that appeared not to be associated with THO in these cellular extracts) (Fig. 1A, cf. lanes 4). Importantly, other well-defined, conspicuous protein bands appeared in the eluted sample (Fig. 1A, left panel, lane 4). Identification of some of the most prominent bands in the Coomassie-stained SDS-PAGE gel by peptide mass fingerprinting (Supplemental Fig. S1) revealed the presence of known nuclear mRNA-binding proteins; namely, the 5′ cap-binding protein Cbp80 and the 3′ poly(A) tail-binding proteins Nab2 and Pab1 [with a relative abundance reflecting the oligomerization of the poly(A)-binding proteins in comparison with the cap-binding complex]. In addition, other nuclear proteins appeared to specifically copurify, such as Yhs7 (an as yet uncharacterized protein encoded at locus *YHR127W*). The most remarkable feature, however, was the striking overstoichiometric representation of Yra1 in the eluted fraction (Fig. 1A, left panel, lane 4; Supplemental Fig. S1). We concluded that the optimized enrichment strategy was efficient in isolating supramolecular THO–Sub2-containing assemblies that have the hallmark proteins expected for nuclear mRNPs.

### Isolated supramolecular assemblies are bona fide nuclear mRNPs

Next, we assessed whether the purified assemblies contain RNA, as would be expected for mRNPs. Upon treatment with the RNA-cleaving endonucleases RNase A/T1 prior to the elution step, the majority of mRNA-binding proteins were indeed released in the flowthrough (Fig. 1B, cf. lanes 1 and 4). Only the THO subunits, a seemingly substoichiometric amount of Sub2, and a small fraction of the initial Yra1 protein remained in the RNase-treated eluted sample (Fig. 1B, cf. lanes 1 and 2). We proceeded to identify the mRNAs associated with the purified assemblies by performing short-read RNA sequencing. Libraries were prepared with RNA extracted from the purified assemblies eluted in a native form with biotin and from total lysate as control. The mRNA population was enriched by affinity-based rRNA depletion, as this strategy is compatible with incomplete 3′ ends.

**Figure 2.**
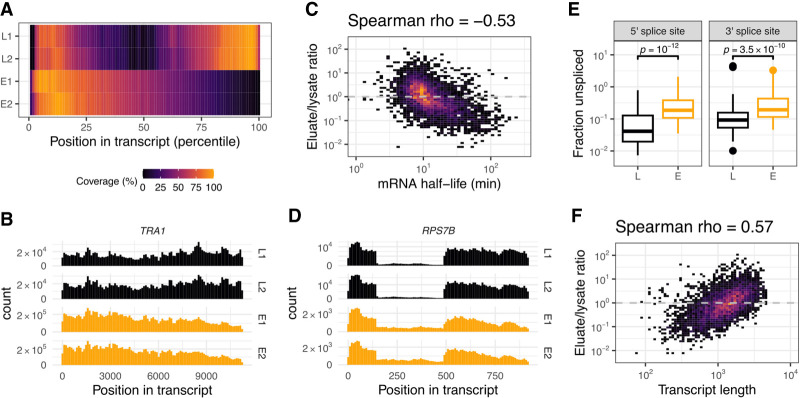
RNA content characterization of purified mRNPs. (*A*) Metagene coverage analysis of transcripts for both RNA-seq replicates, each originating from total lysate (L1 and L2) or natively eluted particles (E1 and E2). Sequence coverage for each annotated yeast transcript open reading frame (nuclear genome-encoded and non-intron-containing) was split into 100 bins and scaled linearly between 0 and 1. Average per bin was further scaled linearly between 0% and 100%. (*B*) Coverage histogram example for a transcript derived from the *TRA1* locus. Samples are as in *A*; histograms have 100 bins. (*C*) Transcript enrichment in eluate over lysate versus previously published mRNA half-lives (Sun et al. 2013). Spearman correlation coefficient is indicated at the *top*. (*D*) Coverage histogram as in *B* for a transcript originating from the monointronic gene *RPS7B*. (*E*) Distribution of the unspliced fraction per transcript for annotated splice sites in the lysate (L) and eluate (E) samples. The fraction of unspliced reads was calculated as the ratio of nonsplit read versus split read counts for reads that overlapped with the annotated splice site (junctions covered with <20 reads were excluded). *P*-values were calculated using the Wilcoxon rank-sum test. (*F*) Transcript enrichment in eluate over lysate versus annotated transcript length for the same subset of transcripts as in *C*.

RNA-seq data from two biological replicates exhibited a high reproducibility, with metagene coverage analysis showing similar patterns within eluates and within lysates, and consistent differences between them (Fig. 2A; Supplemental Fig. S2A). In contrast to the lysates, the mRNA sequence coverage of the eluates showed a 5′ bias (Fig. 2A), indicating the presence of nascent transcripts. When examining individual profiles, however, the bias was not as dramatic as the heat map would suggest (due to normalization of the coverage values). This holds true even in the case of very long transcripts, where unfinished transcription was more evident (such as *TRA1*) (Fig. 2B). Enrichment analysis to assess differences in the abundance of individual transcripts between the lysate and eluate revealed the presence of a nonrandom pool in the plethora of mRNAs present in the eluate. First, mitochondrial-encoded RNAs were highly depleted in the eluted fraction (Supplemental Fig. S2B). Second, we observed a negative correlation with mRNA half-lives: Transcripts known to have short half-lives (Sun et al. 2013) were disproportionally enriched in the eluted samples (Fig. 2C) and thus are unlikely to derive from the cytoplasmic population. Supporting the notion that the purified assemblies correspond to the nuclear population, we detected the presence of reads originating from intronic regions (Fig. 2D). To examine the splicing status of the transcripts, we analyzed the reads overlapping with annotated 5′ and 3′ splice sites. When calculating the ratio of nonsplit reads versus split reads, we found that the unspliced fraction was higher in our isolated particles as compared with the lysate (Fig. 2E). These observations suggest that splicing termination was not required to assemble the particles that we purified via THO and Sub2. Finally, we found a positive correlation for longer transcripts (Fig. 2F; Supplemental Fig. S2B), which appeared to be independent from the half-life correlation (Supplemental Fig. S2C). We attributed the propensity for longer transcripts to a more efficient affinity purification due to the presence of more Sub2 or THO complexes on longer mRNAs. Interestingly, similar observations of Sub2 and Yra1 being enriched on longer transcripts had been reported from in vivo data by PAR-CLIP (Baejen et al. 2014). Thus, the biochemical purification appears to reflect a biologically relevant state of nuclear mRNPs.

### Protein landscape in the nuclear mRNP population

**Figure 3.**
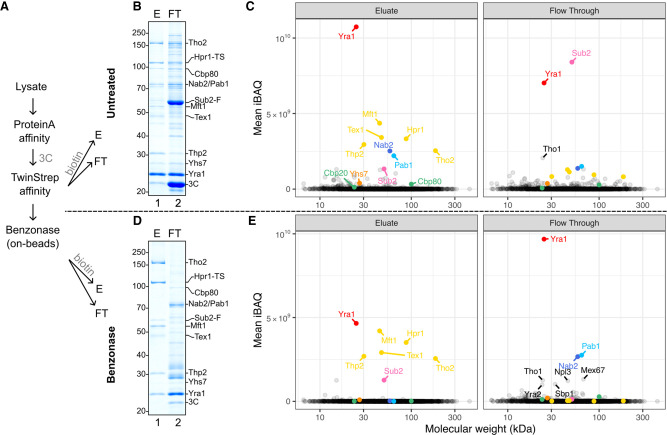
Protein content characterization of purified mRNPs. (*A*) Schematic of sample preparation procedure for quantitative proteomics mass spectrometry analysis from the *hpr1-TS/Sub2-FLAG-3C-ProtA* yeast strain (where Sub2 and Hpr1 served as bait proteins in the affinity purification). Four samples for each condition were prepared by TCA precipitation of native eluates (E) or flowthrough (FT). One sample was used for SDS-PAGE control, and three were used for mass spectrometry analysis. (*B*) Coomassie-stained 10% SDS-PAGE gels of TCA-precipitated native eluate (E) or unbound fractions (FT) from the second affinity step. (*C*) Mean intensity-based absolute quantitation (iBAQ) values from three replicates in each condition from *B*. Each point represents one identified protein positioned horizontally according to its theoretical molecular weight. THO complex components are in yellow, cap binding components are in shades of green, tail binding components are in shades of blue, Sub2 is in pink, Yra1 is in red, Yhs7 is in orange, and additional mRNP-associated proteins are annotated in black. (*D*) Coomassie-stained 10% SDS-PAGE gels of TCA-precipitated native eluate (E) or released fraction (FT) after on-bead benzonase treatment prior to elution from the second affinity step. (*E*) Mean iBAQ values from three replicates in each condition from *D*. Color scheme is the same as in *C*.

To gain a comprehensive view of the protein landscape in the purified assemblies and their relative abundance, we set out to obtain quantitative proteomic data on the samples eluted in native conditions (Fig. 3). The proteolytic digestion protocols were optimized to account for the features of mRNA binding proteins, which often contain extensive intrinsically disordered regions (IDRs) interspersed with positively charged Lys/Arg residues. Quantitation of the label-free proteomic data was carried out in triplicates using intensity-based absolute quantification (iBAQ) (Schwanhäusser et al. 2011). The proteomic results we obtained for the eluted mRNP specimens (Fig. 3A,B, lane 1) demonstrated a marked enrichment of Yra1 as compared with the THO components (Fig. 3C, left panel). Analysis of the flowthrough fraction (Fig. 3B, lane 2) uncovered significant amounts of Sub2 and Yra1, which were either not associated with mRNPs or exceeded the binding capacity of the beads (Fig. 3C, right panel).

Next, we tried to isolate an endogenous RNA-bound THO–Sub2–Yra1-containing unit in the mRNPs. By adding benzonase to the beads prior to the last elution step, we could digest the initial nucleic acid content, although some RNA fragments remained protected (Supplemental Fig. S3). Proteomic analysis of this sample (Fig. 3A,D, lane 1) showed the presence of THO complex proteins Sub2 and Yra1, with the latter in significantly lower amounts as compared with intact mRNPs (Fig. 3C,E, cf. left panels). Analysis of the flowthrough (Fig. 3D, lane 2) revealed that a large amount of Yra1 as well as other mRNA-binding proteins had been released upon benzonase treatment (Fig. 3E, right panel). The results of the quantitative proteomic analysis of the mRNP specimens eluted in native conditions (Fig. 3) were thus consistent with the visual interpretation of the SDS-PAGE analyses of the samples eluted in denaturing conditions (Fig. 1A). While we did not detect spliceosome components above background in our mass spectrometry data, it is possible that they are simply too low in abundance given the scarcity of intron-containing transcripts in our eluates. In general, the presence of overstoichiometric Yra1 and substoichiometric Sub2 in the samples was unexpected in the context of the current view of TREX–mRNP complexes.

### Nuclear mRNP populations show a variety of compact, irregular particles

**Figure 4.**
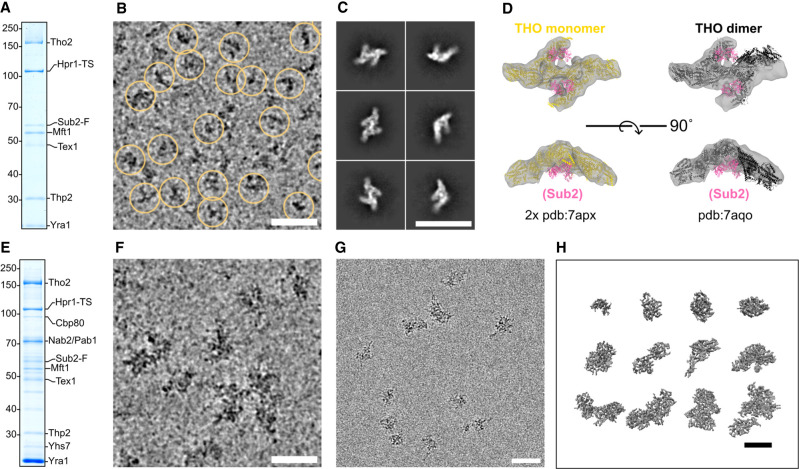
Morphology of isolated nuclear mRNPs by cryo-EM and cryo-ET. (*A*) Coomassie-stained 10% SDS-PAGE gel of a TCA-precipitated aliquot from the benzonase-treated native eluate that was used to prepare the grids in *B*. (*B*) Cryo-electron micrograph crop-out of benzonase-treated particles on a 2-nm carbon-supported grid. Yellow circles indicate picked particles used in the final 3D reconstructions. Acquired at 3-µm defocus. Scale bars in this and all other panels, 500 Å. (*C*) Cryo-EM single-particle analysis. Selected 2D class averages of particles picked from the same specimen as in *B* are shown. (*D*) 3D reconstruction after classification and nonuniform refinement (most populated class out of three) displayed as a gray volume. (*Left*) Two monomers from a previously published structure (Schuller et al. 2020) of recombinant THO–Sub2 are separately fitted in each arm of the volume. THO is yellow, and Sub2 is pink. (*Right*) The dimeric form of THO–Sub2 as observed in the same published structure (THO dimer in black). (*E*) Coomassie-stained 10% SDS-PAGE gel of a TCA-precipitated aliquot from the native eluate that was used to prepare the grids in *F*. (*F*) Cryo-electron micrograph crop-out of native particles on a 2-nm carbon-supported grid. Acquired with a defocus of 3 µm. (*G*) Cryo-electron micrograph crop-out of native particles in free ice. Acquired at 3-µm defocus. (*H*) Selected isolated particles extracted from a reconstructed tomogram after denoising. Acquired at 5-µm defocus.

We proceeded to visualize both the intact mRNPs and the benzonase-treated samples by cryo-EM methods. The low concentration of the native mRNPs generally gave rise to a small number of particles on unsupported cryo-EM specimens. Since attempts to overcome this resulted in an apparent mRNP disassembly (Supplemental Fig. S4A), we used carbon-supported grids for cryo-EM data collection. The cryo-EM data of the benzonase-treated specimen (Fig. 4A,B) showed a sufficiently homogeneous population of particles to proceed with single-particle analysis (Fig. 4C; Supplemental Fig. S4B). This resulted in a reconstruction consistent with the structured core of the dimeric THO complex (THO_core_) but in a more symmetric configuration than previously observed on recombinant THO_core_–Sub2 complexes (Fig. 4D; Schuller et al. 2020; Xie et al. 2021). No density corresponding to Sub2 was visible in the reconstruction (Fig. 4D; Supplemental Fig. S4C). Using similar EM grid preparations, the intact mRNP population (Fig. 4E) appeared as larger particles with heterogeneous size and shape (Fig. 4F). Consequently, the averaging procedures of single-particle cryo-EM yielded no interpretable 2D classes. Template matching approaches also failed to reveal THO complexes despite the fact that they were present in the intact particles (Fig. 4A–D). This is possibly due to THO being too small in size relative to the particles or being partially inside these dense mRNPs.

To overcome the limitations in the cryo-EM analysis of mRNPs, we turned to cryo-electron tomography (cryo-ET). We could obtain few unsupported specimens with enough particles in free ice (Fig. 4G) to collect a tomogram that showed clearly visible particles without denoising and that could be reconstructed (Supplemental Fig. S4D). Differently shaped, compact particles were identified in the reconstruction (Fig. 4H). We emphasize that the samples we imaged were neither cross-linked (to exclude artificial compaction) nor nuclease-treated (to exclude artificial trimming). We concluded that the compactness of the particles in the cryo-ET analysis is a bona fide feature of native mRNPs.

During the revision of our article, a parallel study investigating human mRNPs purified via an affinity tag on the Hpr1 ortholog THOC1 was published (Pacheco-Fiallos et al. 2023). Remarkably, this study showed that human nuclear mRNPs also exist as compact particles. However, there are notable differences. First, the human assemblies exhibited greater uniformity in size and shape as compared with the yeast mRNPs in our study. Second, the human particles presented recognizable THO–UAP56 complexes on their surface. It is currently unclear whether these differences arise, for example, from variations across species or from variations in the sample preparation procedures (such as the nuclease treatment and Grafix cross-linking treatment prior to imaging of the human mRNP samples).

### Protein proximity network in the nuclear mRNPs

**Figure 5.**
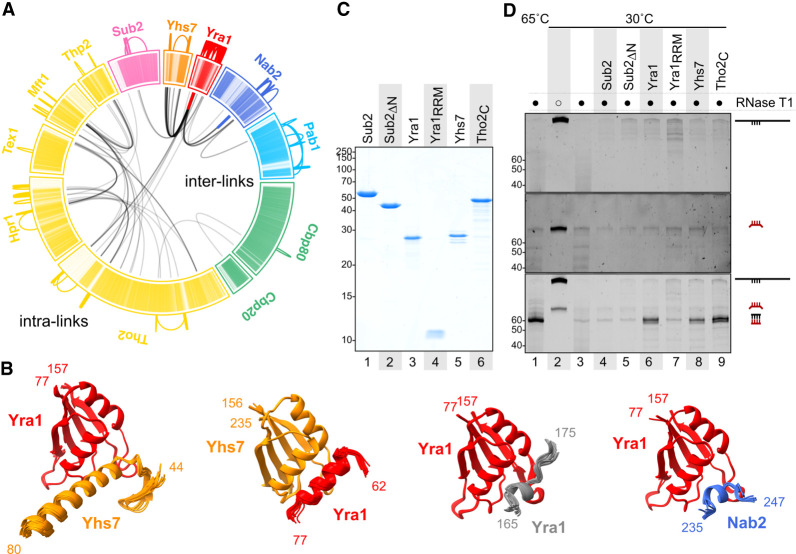
Protein and RNA interaction networks in nuclear mRNPs. (*A*) Location of BS^3^ cross-links in native particles as identified by mass spectrometry. Links between peptides within the same protein (intralinks) are shown in color on the outer region. Links between peptides originating from different proteins (interlinks) are shown in black inside the circle. Inside the circle, colored interlinks represent cross-linking between two copies of the same protein. The sequence of each protein is schematized in individual sectors and colored per protein type (as in Fig. 3C,E). In each protein sector, each line represents a residue (N terminus to C terminus, clockwise) with color intensities corresponding to scaled pLDDT values (AlphaFold per-residue confidence metric for individual predictions). Low pLDDT (which correlates with disorder) is represented in lighter shades. Darker shades (higher pLDDT) correlate well with folded regions. (*B*) AlphaFold predictions of protein fragments selected around identified cross-links in *A*. Twenty-five models predicted with AlphaFold were aligned on the RRM domains of the proteins indicated. Only domains that converged to similar structural features are displayed. Shown are 19 out of 25 models for Yra1_RRM–Yhs7, 20 out of 25 models for Yhs7_RRM–Yra1, 25 out of 25 models for Yra1_RRM–Yra1, and 15 out of 25 models for Yra1_RRM–Nab2. (*C*) Purified recombinant proteins separated on a 15% Coomassie-stained SDS-PAGE used as input in an RNA-annealing assay (shown in *D*). (*D*) RNA-annealing assay. A 315-nt in vitro transcribed RNA substrate derived from HHF1 mRNA (in black) was mixed with an 80-nt probe (in red). The probe contained a 60-nt sequence of perfect complementarity to part of the substrate flanked by 10-nt nonpairing extensions on either side. After incubation at the indicated temperatures with the corresponding proteins, treatment with RNase T1 digested all unpaired fragments. The remaining RNA was separated on a 14% urea-PAGE and stained with SYBR Gold. (*Top*) Substrate alone. (*Middle*) Probe alone. (*Bottom*) Both substrate and probe present.

For nuclear mRNPs to be highly compacted, the proteins contained within the particles would be expected to be positioned in close proximity to each other. To address this, we used cross-linking mass spectrometry (XL-MS), an approach that is compatible with structurally and compositionally heterogenous complexes (O'Reilly and Rappsilber 2018). The mRNP preparation was subjected to XL-MS with the bifunctional cross-linker bis(sulfosuccinimidyl)suberate (BS^3^) (Supplemental Fig. S5A). We used a workflow tailored to the characteristic features of mRNP proteins (for example, using diluted samples to avoid cross-reaction of the Lys-containing IDRs between different particles). The XL-MS data we obtained had a balanced number of intralinks (125 cross-links in which both ends of BS^3^ reacted within the same protein) and interlinks (95 cross-links in which the two ends of BS^3^ connected two different proteins) (Fig. 5A). As a control, analysis of modified peptides where one end of BS^3^ reacted with water or Tris buffer showed a comprehensive coverage of lysine residues that were accessible to the cross-linking reagent in the reaction (335 monolinks) (Supplemental Fig. S5B).

The XL-MS data had high information content. The intralinks (which could also occur within homo-oligomeric species) were consistent with the known structure of THO_core_ (Supplemental Fig. S5C). Importantly, the intralinks also showed additional features. First, extensive intralinks were present for the poly(A)-binding proteins Pab1 and Nab2, in line with their known RNA-dependent homo-oligomerization properties (Fasken et al. 2019; Schäfer et al. 2019). Second, the XL-MS data showed a striking hotspot of intralinks in Yra1 (Fig. 5A), suggesting that multiple copies of Yra1 are in spatial proximity in the mRNPs. Supporting this interpretation, two intralinks were identified between either overlapping peptides or identical lysines of Yra1 molecules, and the same occurred in the case of Nab2 (Fig. 5A, thick red and blue lines in the interlink part of the diagram), indicating that the same regions of these molecules are close to each other.

The analysis of the interlinks also showed good agreement with the known structures of THO_core_, Sub2, and Cbp80–Cbp20 (Supplemental Fig. S5C). No interlinks were detected between THO_core_ and Sub2, in line with the absence of density features in the endogenous benzonase-treated reconstructions (Fig. 4D). Notably, Yra1 showed an extensive network of interlinks with Nab2, with the C terminus of Tho2, and in particular with Yhs7 (Fig. 5A). We concluded that in the compact mRNP particles, multiple copies of Yra1 are in spatial proximity to each other, to Yhs7, to the Tho2 C terminus, and to Nab2.

### Protein–protein interaction nodes in the mRNP network

We harnessed the XL-MS information to guide and assess predictions of physical protein–protein interactions between mRNP network proteins by AlphaFold (Jumper et al. 2021). Yra1 and Yhs7 have a similar overall domain organization (Supplemental Fig. S5D), with an RRM (RNA recognition motif) domain and prominent IDRs interspersed with small helical segments. The C terminus of Tho2 lacks an RRM domain but also features an extensive IDR. To directly visualize whether the chemically modified residues in the XL-MS experiments occurred in structured or unstructured regions of the corresponding proteins, the per-residue confidence scores of individual AlphaFold predictions (high for structured regions and low for unstructured regions) were incorporated in the cross-linking maps (in dark and light color shades, respectively) (Fig. 5A). From the mapping, the interlinks appeared to cluster mostly in structured regions of Yra1, Yhs7, and Nab2.

Focusing on the interlinked regions and using them as input to AlphaFold-Multimer (Evans et al. 2021), an extension of AlphaFold developed for protein–protein complexes, we obtained computational predictions of Yra1–Yhs7 and Yra1–Yra1 interactions that converged to stable structures (>15 out of 25 models), consistent with experimental interlinks (Fig. 5B). Similar stable predictions were obtained for the interactions between Yra1 and Nab2 (Fig. 5B). In general, the predicted protein–protein interactions occurred between the RRM domain of Yra1 and short helical elements in Yhs7 and Nab2, and similarly between the RRM domain of Yhs7 and a small helical segment of Yra1 (Fig. 5B). Despite involving rather small surface areas, the predicted interactions centered on hydrophobic residues (with an apolar surface at the helical side of the RRM domains). The predictions were not indiscriminate. For example, in the case of Yra1–Tho2, we did not obtain a convincing structural prediction with AlphaFold. It is possible that segments in the IDRs may also engage in additional interactions; for example, in cross-β-type interactions (Kato and McKnight 2021) or inherently dynamic interactions (Borgia et al. 2018) or their proximity is mediated by RNA.

### mRNP network proteins promote RNA–RNA interactions

The RRM domain of Yra1 is considered noncanonical, as it lacks the RNA-binding capabilities typically associated with canonical RRMs (Zenklusen et al. 2001). Consistent with this, we found that the Yra1 RRM serves as a prominent protein interaction hotspot in the endogenous mRNP particles (Fig. 5A,B). The RNA-binding properties of Yra1 lie in positively charged IDRs (Zenklusen et al. 2001; Keil et al. 2023). The RNA-binding properties of Yra1 underpin its strong RNA–RNA-annealing properties (Portman et al. 1997), an in vitro readout for the capacity to promote RNA–RNA interactions. Besides Yra1, the IDRs in Yhs7 and in the C terminus of Tho2 are also rich in positively charged residues, and in the latter case have been shown to harbor nucleic acid-binding properties important for mRNP biogenesis (Peña et al. 2012). These observations raised the possibility that RNA–RNA interaction-promoting properties may not be confined to Yra1 but are shared with other mRNP network proteins. To address this, we expressed and purified Yra1, Yhs7, and the Tho2 C terminus (Fig. 5C) and tested the recombinant proteins in RNA-annealing assays (Fig. 5D), inspired by the original study on Yra1 (Portman et al. 1997). We note that in the context of mRNP assembly, the mRNA condensation process is expected to involve promiscuous binding and adherence of different segments within an mRNA, which will favor annealing if the sequences brought into the vicinity have a certain degree of complementarity. RNA-annealing assays, which monitor the base-pairing of fully complementary RNAs, are a robust biochemical system that serves as a proxy to assess RNA–RNA interactions.

In the RNA-annealing assays, we monitored the base-pairing of two RNAs with a stretch of 60 complementary nucleotides by treatment with RNase T1 (which cannot digest double-stranded RNA). At 30°C, the two RNAs were not able to anneal on their own, as judged by the degradation by RNase T1 (Fig. 5D, cf. lanes 2 and 3). As previously reported (Portman et al. 1997), addition of full-length Yra1 resulted in the formation of the annealed product (Fig. 5D, lane 6). Remarkably, both Yhs7 and the Tho2 C terminus showed similarly robust RNA–RNA-annealing activities (Fig. 5D, lanes 8,9). The activity was not indiscriminate, as there was no annealing by adding the Yra1 RRM domain or Sub2 (which contains a negatively charged IDR) (Fig. 5D, lanes 7,4). As a note, other mRNP proteins containing unstructured positively charged IDRs are present in lower amounts in our purified mRNPs (such as Yra2, Npl3, and Sbp1) (Fig. 3E, right panel) and may have similar RNA–RNA interaction-promoting properties, particularly if enriched in specific subpopulations. We concluded that several mRNP network proteins with positively charged tails have the capacity to promote RNA–RNA interactions.

The orthologs of Yra1 and Tho2 in human cells, Aly/REF and THOC2, share a similar domain organization. To address whether they could also promote RNA–RNA interactions, we expressed and purified recombinant human Aly/REF and the human THOC2 C terminus and evaluated them in RNA-annealing assays (Supplemental Fig. S6A). Our findings demonstrate that Aly/REF and the THOC2 C terminus possess RNA-annealing capability, as observed for the yeast orthologs, underscoring the evolutionary conservation of their RNA–RNA interaction-enhancing properties.

## Discussion

**Figure 6.**
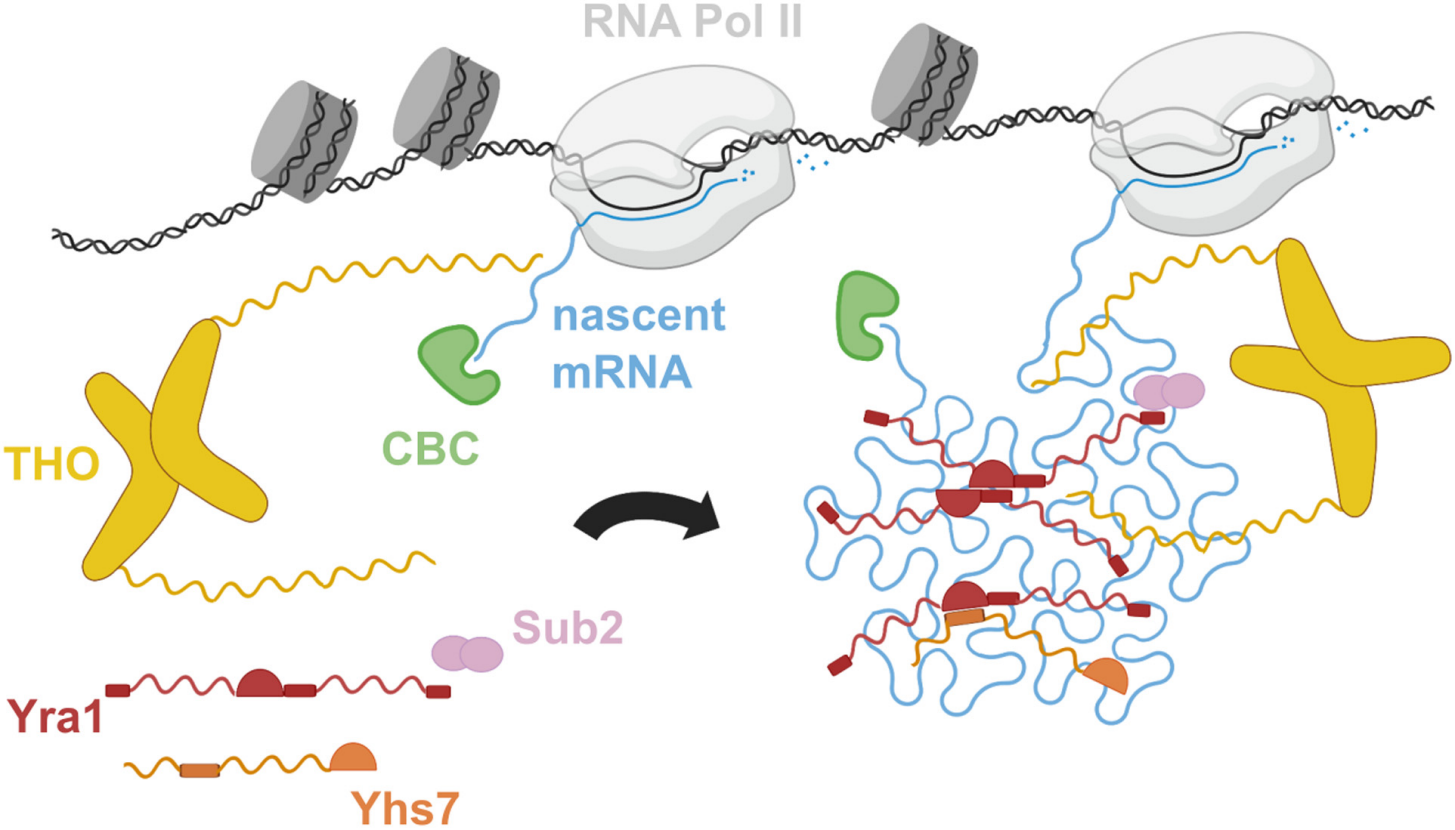
The mRNP IDR network model of nuclear mRNA packaging. Schematic representation of nuclear mRNP cotranscriptional assembly and compaction. The proteins are represented with the colors and architecture described in the text. Nascent mRNA is in cyan.

In this study, we purified native yeast mRNPs and characterized their structural organization by using a range of experimental approaches. We found that yeast nuclear mRNPs are highly compacted particles with an interlaced network of mRNA-binding proteins centered at Yra1, the most abundant protein in these assemblies. Yra1 and other mRNP network proteins, such as Tho2 and Yhs7, have the capacity to promote RNA–RNA interactions via their positively charged IDRs. In addition to their RNA-binding regions, several mRNP network proteins (including Yra1, Yhs7, and Nab2) have structured domains that can engage in physical interactions when brought in close proximity in the mRNP condensation process. We refer to this mechanism as the “IDR network model” of mRNP packaging, where positively charged IDRs extend from protein–protein interaction nodes and promote mRNA compaction (Fig. 6).

The yeast mRNP network proteins Yra1 and Tho2 share many structural and functional similarities with human Aly/REF and THOC2 (Stutz et al. 2000; Infantino and Stutz 2020), including their RNA–RNA interaction-promoting properties (Fig. 5A; Supplemental Fig. S6A). Human Aly/REF is found on both intronless mRNAs (Taniguchi and Ohno 2008) and intron-containing mRNAs (Masuda et al. 2005; Viphakone et al. 2019). In the latter case, it has been found to be associated with the eIF4A3–Mago-Y14 components of the exon–junction complex (EJC) (Le Hir et al. 2000; Gromadzka et al. 2016). Using AlphaFold, we obtained a reproducible structural model of an eIF4A3–Mago-Y14–Aly/REF complex. In this model, the positively charged IDRs of Aly/REF extend flexibly from the structured core (Supplemental Fig. S6B), suggesting that Aly/REF could maintain its ability to enhance RNA–RNA interactions even within the EJC. During the revision of this article, an alternative “EJC oligomerization model” for human mRNP compaction was proposed based on the cryo-EM reconstruction of an eIF4A3–Mago-Y14ΔN–Aly/REFΔC truncation mutant (Pacheco-Fiallos et al. 2023). Notably, AlphaFold predictions of the full-length complex converged on similar interaction sites for Aly/REF within the EJC, albeit in the monomeric form (Supplemental Fig. S6B). Whether and how EJC oligomerization can occur in the context of the full-length complex and in native particles are still unclear, but there may also be other mechanisms by which EJC components participate in mRNP network interactions. In this context, upon analyzing the published cross-linking mass spectrometry data (Pacheco-Fiallos et al. 2023), we noticed a prominent interaction hub between Aly/REF and the proteins CHTOP (Chang et al. 2013; Viphakone et al. 2019) and ERH (Graille and Rougemaille 2020). Guided by these proximity data, we identified a possible Aly/REF–ERH–CHTOP protein–protein interaction module with protruding positively charged IDRs using AlphaFold predictions (Supplemental Fig. S6C). Notably, this module is compatible with Aly/REF also when it is associated with EJC proteins (Supplemental Fig. S6C). More generally, the Aly/REF–ERH–CHTOP module in human mRNPs could conceptually correspond to the Yra1–Yhs7 module in the yeast mRNP network.

The IDR network model raises a general question: Considering the natural propensity of ribonucleotide chains to fold and compact by base-pairing within themselves (Schultes et al. 2005), why would mRNP network proteins be required? First, packaging with mRNP network proteins (such as Yra1 and the THO complex scaffold subunit Tho2) prevents tangling of the nascent transcript in R-loops. Second, spontaneous high-order compaction of a long, negatively charged polymer like mRNA is unlikely to occur unaided in cellular conditions (such as at physiological salt and magnesium concentrations). mRNP network proteins with basic IDRs could act as flexible macromolecular counterions, reminiscent of the mode of action proposed for the hepatitis C virus nucleocapsid protein in assisted viral RNA folding (Holmstrom et al. 2019). Finally, condensation without proteins could also lead to collapsed, kinetically trapped states that would be difficult to remodel, as local RNA structure is quite stable. Organized condensation around the positively charged IDRs of mRNP network proteins offers a more reversible structural order that can also be regulated by post-translational modifications. Indeed, lysine ubiquitylation of yeast Yra1 and arginine methylation of Aly/REF have been shown to regulate handover mechanisms at the nuclear export step (Hung et al. 2010; Iglesias et al. 2010). The “IDR network” model (Fig. 6) provides a molecular framework for the cotranscriptional compaction of nascent mRNAs, enabling the organized, regulatable, and reversible packaging of nuclear mRNP particles. This model serves as a versatile and universal mRNP-packaging mechanism applicable to both intron-containing and intronless transcripts across various organisms from yeast to humans.

## Materials and methods

### Endogenous nuclear mRNP isolation

*S. cerevisiae* (BY4741 background) was engineered to add C-terminal purification tags at genomic loci using standard yeast genetics techniques. Coding sequences for all tags (Twin-Strep-3C-protein A, FLAG-3C-protein A, and Twin-Strep), separated from the last amino acid in the protein sequence by a (GS)_5_ linker, were amplified by PCR from template vectors using primers including 45- to 50-nt overhangs for homologous recombination. Integration was confirmed by PCR and Western blot.

Yeast cultures were grown in YPD to an absorbance of ∼1 at 600 nm, harvested by filtration, and immediately frozen in liquid nitrogen. Frozen yeast pellets were ground with a cryogenic grinder (SPEX), resuspended in ice-cold purification buffer (50 mM potassium phosphate at pH 8, 0.1% NP40), and incubated for 30 min with protein-G Dynabeads (Thermo Fisher Scientific 10004D) coated with antiprotein A IgG (Sigma-Aldrich P2921). Beads were separated from the lysate with a magnet, washed in purification buffer, resuspended in purification buffer containing 3C protease, and rotated for 30 min at 4°C. 3C eluates were incubated for 30 min at 4°C with epoxy-activated M270 Dynabeads (Thermo Fisher Scientific 14301) previously conjugated to Strep-Tactin (IBA Lifesciences 2-1204-005). Strep-Tactin beads were washed again in purification buffer and eluted either 5 min in SDS-loading dye for SDS-PAGE analysis or 30 min in purification buffer containing 10 mM biotin and NP40 reduced to 0.01% for RNA extraction, mass spectrometry, and cryo-EM/cryo-ET sample preparation.

### RNA sequencing

Total RNA was extracted with the hot acid phenol method (three consecutive extractions with 1-bromo-3-chloropropane; Sigma B9673) from lysate before the first affinity step and from biotin eluates after the last affinity step. Extracted RNA (10 µg) was further treated with Turbo DNase (Thermo Fisher Scientific AM2238) and subjected to RNA Clean & Concentrator kit (Zymo Research R1015). rRNA depletion was performed using riboPOOLs rRNA depletion kit (siTOOLs Biotech 27DP-K006-000005).

Ten nanograms of rRNA-depleted RNAs was used for RNA sequencing library preparation with NEBNext Ultra II directional RNA library preparation kit for Illumina (NEB0 E776). RNAs and libraries were analyzed by a Qubit Flex fluorometer (Thermo Fisher Scientific Q33327) and a 2100 Bioanalyzer instrument (Agilent G2939BA) for sample quality control. Paired-end sequencing was performed on an Illumina NextSeq 500 (75 bp–75 bp). All four samples (L1, L2, E1, and E2) were multiplexed and sequenced in a single Mid-Output 150-cycle kit v2.5 to reduce sequencing batch effects (see also Supplemental Fig. S2).

### Quantitative mass spectrometry

TCA-precipitated samples were solubilized by addition of 50 µL of 1× SDC buffer [1% sodium deoxycholate (SDC), 40 mM 2-chloroacetamide (CAA), 10 mM Tris(2-carboxyethyl) phosphine (TCEP), 100 mM Tris at pH 8] and sonication using a Bioruptor Plus system (Diogenode) 10 times for 30 sec at high intensity. After incubation for 20 min at 37°C, the samples were diluted 1:1 with water and digested overnight at 37°C by addition of 1 µg of LysC (Wako). Next, the solution was acidified with trifluoroacetic (TFA) to a final concentration of 1%, followed by desalting the peptides using SCX stage tips.

Peptides were dissolved in buffer A (0.1% formic acid), and one-third of the peptides were analyzed using a LC-MS/MS setup comprising an Easy-nLC 1200 (Thermo Fisher Scientific) coupled to a QExactive HF mass spectrometer (Thermo Fisher Scientific). Raw data were processed using the MaxQuant computational platform (version 2.0.1.0) (Cox and Mann 2008) with standard settings applied. The iBAQ algorithm was used for calculation of abundances for the identified proteins (see also Supplemental Fig. S1).

### Cross-linking mass spectrometry

TCA-precipitated, BS^3^-cross-linked samples were solubilized by addition of 8 M urea in 50 mM Tris and sonication using a Bioruptor Plus system (Diogenode) 10 times for 30 sec at high intensity. After dilution to 4 M, samples were incubated with 1 mM MgCl_2_ and 1% benzonase to remove RNA. For reduction and alkylation, 10 mM TCEP and 40 mM CAA were added. After incubation for 20 min at 37°C, samples were diluted 1:3 with water and digested overnight at 37°C by addition of 1 µg each of LysC (Wako) and Trypsin (Promega). Next, the solution was acidified with TFA to a final concentration of 1%, followed by desalting of the peptides using SCX stage tips. Desalted peptides were fractionated into eight concatenated fractions using high-pH reversed-phased fractionation.

Peptides were dissolved in buffer A (0.1% formic acid), and half of the peptides were analyzed using a LC-MS/MS setup comprising an Easy-nLC 1200 (Thermo Fisher Scientific) coupled to an Exploris 480 mass spectrometer (Thermo Fisher Scientific). The acquired raw data were processed using Proteome Discoverer (version 2.5.0.400). Trypsin/P was specified as protease, and up to two missed cleavages were allowed. Furthermore, identifications were only accepted with a minimal score of 40 and a minimal Δ score of 4. Filtering at 1% false discovery rate (FDR) at CSM and cross-link levels were applied (see also Supplemental Fig. S5).

### Cryo-grid preparation

Four microliters of biotin eluate was applied to holey carbon grids (R2/1 Cu, 200 mesh, with or without 2-nm ultrathin carbon film; Quantifoil) freshly cleaned by glow discharge with negative polarity at 20 mA for 20 sec using an EMS GloQube (Quorum Technologies). Grids were plunge-frozen in a liquid ethane/propane mix using a Vitrobot Mark IV (Thermo Fisher Scientific) operated at 4°C and 95% humidity.

### Cryo-electron tomography

A tilt series was recorded on a Titan Krios (300-kV acceleration voltage; model G2, Thermo Fisher Scientific) equipped with a Gatan K3 detector operating in electron-counting mode and a GIF Quantum energy filter (10-eV slit width) at a nominal magnification of 42000×, resulting in a 2.154-Å pixel size. Applied defocus was 5 µm, and a 100-µm objective aperture was inserted. Tilts were recorded as eight-frame movies using a dose-symmetric tilt scheme between −60° and +60° in 4° increments for a total dose of 135 e−/Å^2^. Tilts images were binned four times and aligned iteratively using patch tracking. After reconstruction with weighted back projection, the tomogram was denoised with cryo-CARE (Buchholz et al. 2019) using a locally trained model (see also Supplemental Fig. S4).

### Cryo-electron microscopy

Data were collected on a Glacios microscope (200-kV acceleration voltage; Thermo Fisher Scientific) equipped with a Gatan K2 direct electron detector operating in electron-counting mode at a nominal magnification of 22,000×, resulting in a 1.885-Å pixel size. A 70-µm objective aperture was inserted. A selection of 30,248 particles was used for ab initio model generation in two classes, giving one class whose projections matched well with 2D class averages of input particles. Some 2D classes showed the presence of an almost symmetric THO dimer, while other classes had only one monomer clearly defined (due to possibly damaged particles and previously observed conformational variability in the relative orientation of THO monomers). To separate those by 3D classification, we generated one symmetric template by refining the best ab initio model with imposed C2 symmetry and a monomeric template by manually cropping one monomer from this C2-refined volume using ChimeraX. These volumes (one symmetric dimer and two monomers) were low-pass-filtered to 50 Å and used as templates in a heterogeneous refinement step including all accepted particles without any imposed symmetry (see also Supplemental Fig. S4).

### AlphaFold-Multimer predictions

Predictions were performed with AlphaFold (Evans et al. 2021; Jumper et al. 2021) version 2.2.0 using full databases set up by the Max Planck Computing and Data Facility. Modeling was set to “multimer,” with five models and five predictions per model, each using different random seeds, resulting in 25 predicted structures. Resulting models were aligned with ChimeraX (Pettersen et al. 2021) to assess prediction convergence. Full-length protein sequences were used as input for Yra1_RRM–Yhs7. For the other proteins, regions containing converging predictions were selected and submitted again; namely, Yra1(62–157)–Yhs7(156–243), Yra1(77–157)–Yra1(157–201), and Nab2(235–247)–Yra1(77–157).

### Recombinant protein purification

DNA sequences coding for *S. cerevisiae* Sub2 full-length, Sub2ΔN(50–446), Yra1 full-length, Yra1RRM(76–160), Yhs7 full-length, and Tho2C(1221–1597) were cloned in a bacterial expression vector fused at their 5′ ends to a sequence coding for a His-SUMO tag cleavable with SENP2 protease. Proteins were expressed in BL21(DE3) STAR pRARE (Stratagene) in Terrific broth (TB) medium. Cells were mixed; resuspended in lysis buffer (50 mM sodium phosphate at pH 7.5, 250 mM NaCl, 40 mM imidazole, 5 mM β-mercaptoethanol) supplemented with 5 mg/mL DNase, 5 U/mL benzonase, 5 mM MgSO_4_, and 1 mM PMSF; and lysed by sonication. All constructs were purified using nickel-based affinity chromatography followed by tag cleavage with SENP2 for 15 h at 4°C. The Sumo tag and the protease were removed by a reverse nickel affinity step. In the case of Yra1, Yhs7, and Tho2C, an additional purification step was added to remove remaining nucleic acids: After a 15-fold dilution in buffer A (20 mM Tris at pH 8.0, 100 mM NaCl, 2 mM DDT), the proteins were bound to a heparin chromatography column (Cytiva) and eluted within a 20-CV gradient of buffer B (20 mM Tris at pH 8.0, 1 M NaCl, 2 mM DDT). For all proteins, a final size exclusion chromatography step was performed (Superdex 200) in size exclusion buffer (20 mM HEPES at pH 7.5, 200 mM NaCl, 2 mM DDT or 20 mM HEPES at pH 7.5, 350 mM NaCl, 2 mM DDT) for Yra1.

### RNA-annealing assay

Template for substrate (coding sequence of histone H4 [HHF1] mRNA open reading frame) was amplified from *S. cerevisiae* genomic DNA by PCR with a 5′-oligo providing a T7 RNA polymerase promoter sequence. Template for probe transcription was obtained by annealing synthetic oligos (Sigma-Aldrich) for T7 promoter and a 60-nt fragment with perfect complementarity to HHF1 flanked by 10-nt noncomplementary overhangs on either side. RNA was transcribed in vitro and gel-purified after migration on an 8% acrylamide/7 M urea gel (see also Supplemental Fig. S6A).

Substrate and/or probe (1 pmol of each) were mixed with 10 pmol of protein in a 10-µL reaction mix with a final buffer composition of 20 mM HEPES (pH 7.5), 100 mM KCl, 20 mM NaCl, 0.5 mM magnesium diacetate, and 0.5 mM DTT. After a 10-min incubation at 30°C, 1 U of RNase T1 (Thermo Fisher Scientific) was added, and the mixtures were further incubated for 15 min at 37°C before phenol–chloroform–isoamyl alcohol (Thermo Fisher Scientific) extraction and ethanol precipitation. RNA pellets were resuspended in loading buffer (70% formamide, 7 mM EDTA, 0.07% orange G), heated for 5 min at 70°C, and separated on a 14% polyacrylamide (19:1 acrylamide:bisacrylamide)/7 M urea gel in 1× TBE. The gel was stained for 10 min with SYBR Gold in 1× TBE and imaged under UV illumination with a Gel Doc EZ imager (Bio-Rad).

### Data analysis and representation

Statistical analyses and plotting were performed with R using the tidyverse collection of packages (Wickham et al. 2019). Bioconductor package DESeq2 (Love et al. 2014) was used for RNA-seq data enrichment analysis. ChimeraX (Pettersen et al. 2021) was used to prepare all 3D structure-related figures.

### Data and code availability

RNA-seq data have been deposited in GEO with accession number GSE226854. Mass spectrometry data have been deposited to the ProteomeXchange Consortium (http://proteomecentral.proteomexchange.org) via the PRIDE partner repository with the data set identifier PXD040736. The EM map has been deposited in the EM database with accession number EMD-16841.

## Supplementary Material

Supplemental Material
