# Supplementary material for: Nuclear mRNPs are compact particles packaged with a network of proteins promoting RNA–RNA interactions
Source: Genes Dev. 2023 Jun 1;37(11-12):505–17. doi: 10.1101/gad.350630.123 (PMC10393194; doi:10.1101/gad.350630.123)
Supplement: Supplemental Material [file supp_37_11-12_505__DC1.html]

Nuclear mRNPs are compact particles packaged with a network of proteins promoting RNA–RNA interactions — Nuclear mRNPs are compact particles packaged with a network of proteins promoting RNA–RNA interactions — Supplemental Material 

# Nuclear mRNPs are compact particles packaged with a network of proteins promoting RNA–RNA interactions

## Supplemental Material

- Supplemental\_Fig\_S1.pdf
- Supplemental\_Fig\_S2.pdf
- Supplemental\_Fig\_S3.pdf
- Supplemental\_Fig\_S4.pdf
- Supplemental\_Fig\_S5.pdf
- Supplemental\_Fig\_S6.pdf
