# Supplementary material for: Nuclear mRNPs are compact particles packaged with a network of proteins promoting RNA–RNA interactions
Source: Genes Dev. 2023 Jun 1;37(11-12):505–17. doi: 10.1101/gad.350630.123 (PMC10393194; doi:10.1101/gad.350630.123)
Supplement: Supplemental Material [file supp_gad.350630.123_Supplemental_Fig_S1.pdf]

Supplemental Figure S1

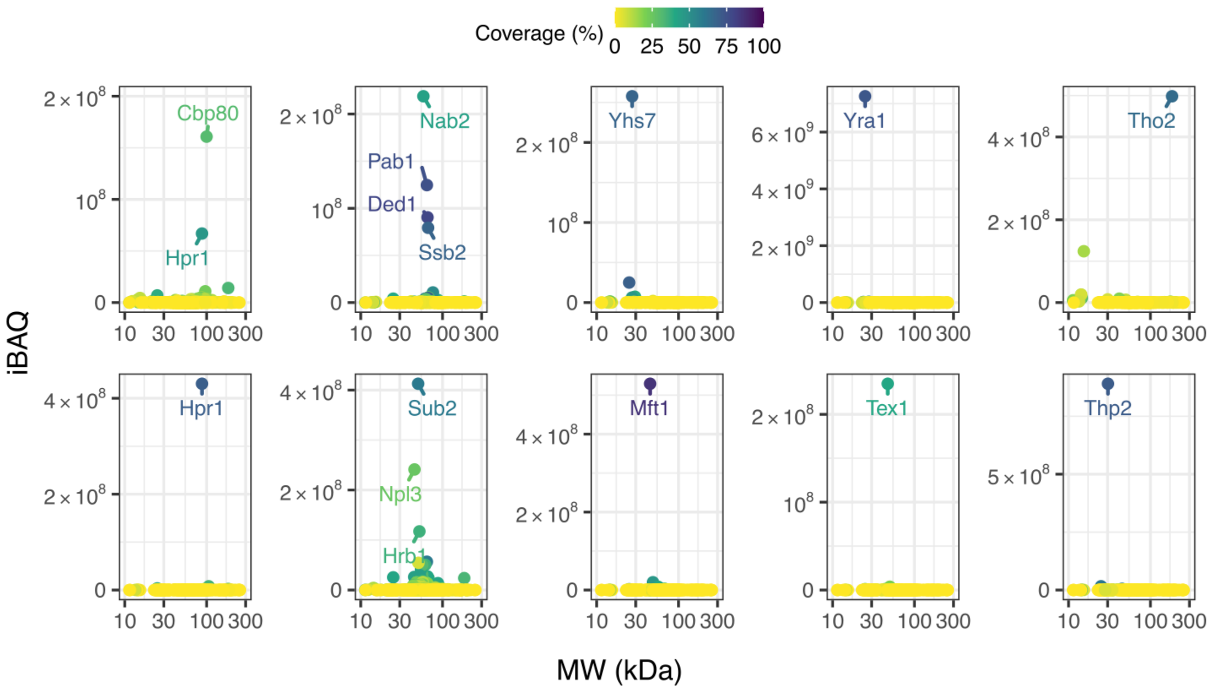

Peptide mass fingerprinting analyses

Intensity-based absolute quantitation (iBAQ) of proteins identified in selected bands cut-out from the gels in Fig. 1 A. Each point represents one identified protein positioned horizontally according to its theoretical molecular weight (MW) and colored according to the sequence coverage of all detected peptides attributed to this protein.

For these analyses, peptides were separated at a flow rate of 250 nL/min by a gradient of buffer B (80% acetonitrile, 0.1% formic acid) from 8% to 30% with an additional increase to 60%. The analytical column (30 cm, inner diameter: 75 microns; packed in-house with ReproSil-Pur C18-AQ 1.9-micron beads, Dr. Maisch GmbH) was heated to 60°C. The mass spectrometer was operated in data-dependent mode with survey scans from m/z 300 to 1650 Th (resolution of 60k at m/z = 200 Th), and up to 10 of the most abundant precursors were selected and fragmented using Higher-energy C-trap Dissociation (HCD with a normalized collision energy value of 28). Normalized AGC targets for MS1 and MS2 scans were set to 3 x 10^6 and 1 x 10^5, respectively, with a maximum injection time of 100 ms for the MS1 scans and 60 ms for MS2 scans.

The peak list was searched against the Uniprot database of *S. cerevisiae* of with an allowed precursor mass deviation of 4.5 ppm and an allowed fragment mass deviation of 20 ppm. MaxQuant enables individual peptide mass tolerances by default and was applied in the search. Cysteine carbamidomethylation, methionine oxidation and N-terminal acetylation were set as variable modifications. LysC was set as protease. The match-between-run option was enabled.
