# Supplementary material for: Nuclear mRNPs are compact particles packaged with a network of proteins promoting RNA–RNA interactions
Source: Genes Dev. 2023 Jun 1;37(11-12):505–17. doi: 10.1101/gad.350630.123 (PMC10393194; doi:10.1101/gad.350630.123)
Supplement: Supplemental Material [file supp_gad.350630.123_Supplemental_Fig_S2.pdf]

Supplemental Figure S2

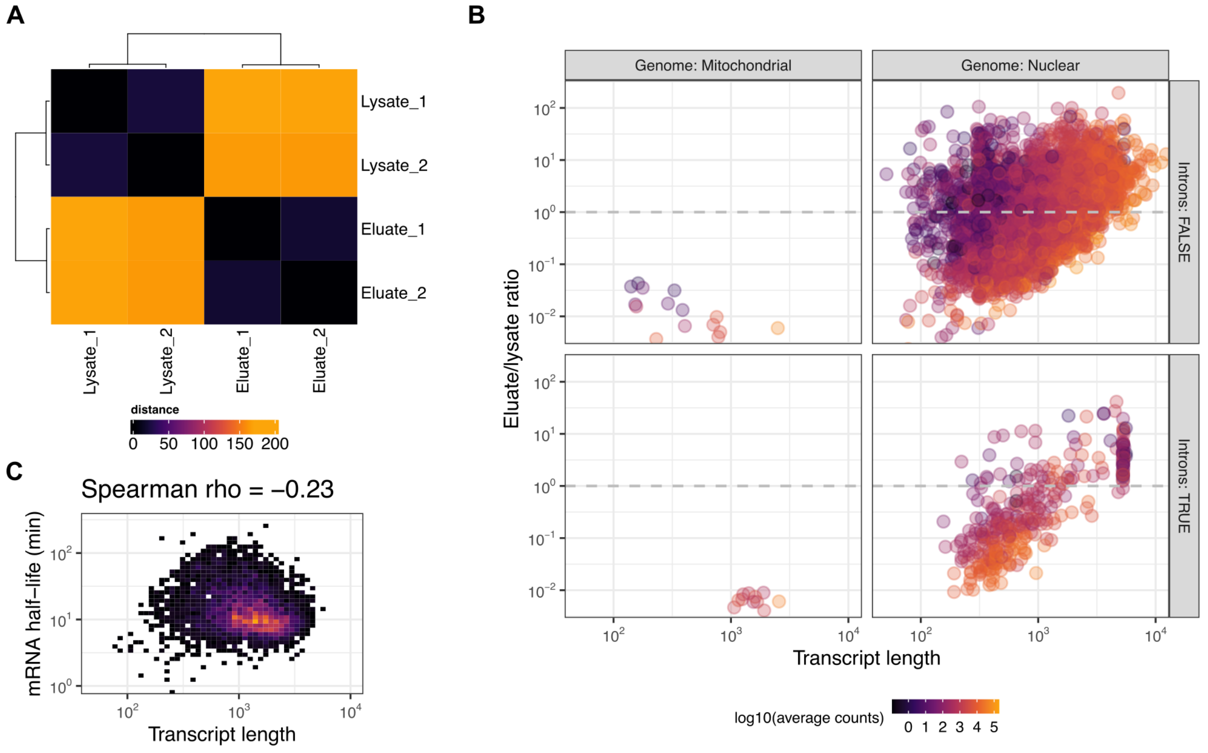

RNA sequencing analyses

A) Heatmap of the sample-to-sample euclidian distances using regularized log-transformed RNAseq count data.

B) Transcript enrichment in eluate over lysate versus annotated transcript length. Transcripts are faceted by their genome of origin (mitochondrial or nuclear) and by the presence of at least one annotated intron within. Each point represents one transcript, colored according to its average abundance in the whole dataset (log10-transformed average counts).

C) Previously published mRNA half-lives (Sun et al. 2013) versus annotated transcript length.

For these analyses, BCL raw data were converted to FASTQ format and demultiplexed with bcl2fastq conversion software (Illumina), which generated 18-35 million reads per samples. Reads were aligned to the sacCer3 genome with STAR (2.7.9a (Dobin et al. 2013) and counts per transcripts obtained with featureCounts (Subread 2.0.3 (Liao et al. 2013)) using the sacCer3 sgdGene annotations, counting paired-end fragments, excluding chimeric pairs and pairs where ends did not align. Scaled bigWig files for coverage analysis and display were generated with deeptools (3.5.1 (Ramírez et al. 2016)) at 1-nt resolution using scaling factors obtained with the DESeq2 analysis.

Dobin A, Davis CA, Schlesinger F, Drenkow J, Zaleski C, Jha S, Batut P, Chaisson M, Gingeras TR. 2013. STAR: ultrafast universal RNA-seq aligner. *Bioinformatics* **29**: 15–21.

Liao Y, Smyth GK, Shi W. 2013. The Subread aligner: fast, accurate and scalable read mapping by seed-and-vote. *Nucleic Acids Res* **41**: e108.

Ramírez F, Ryan DP, Grüning B, Bhardwaj V, Kilpert F, Richter AS, Heyne S, Dündar F, Manke T. 2016. deepTools2: a next generation web server for deep-sequencing data analysis. *Nucleic Acids Res* **44**: W160–5.
