# Supplementary material for: Nuclear mRNPs are compact particles packaged with a network of proteins promoting RNA–RNA interactions
Source: Genes Dev. 2023 Jun 1;37(11-12):505–17. doi: 10.1101/gad.350630.123 (PMC10393194; doi:10.1101/gad.350630.123)
Supplement: Supplemental Material [file supp_gad.350630.123_Supplemental_Fig_S3.pdf]

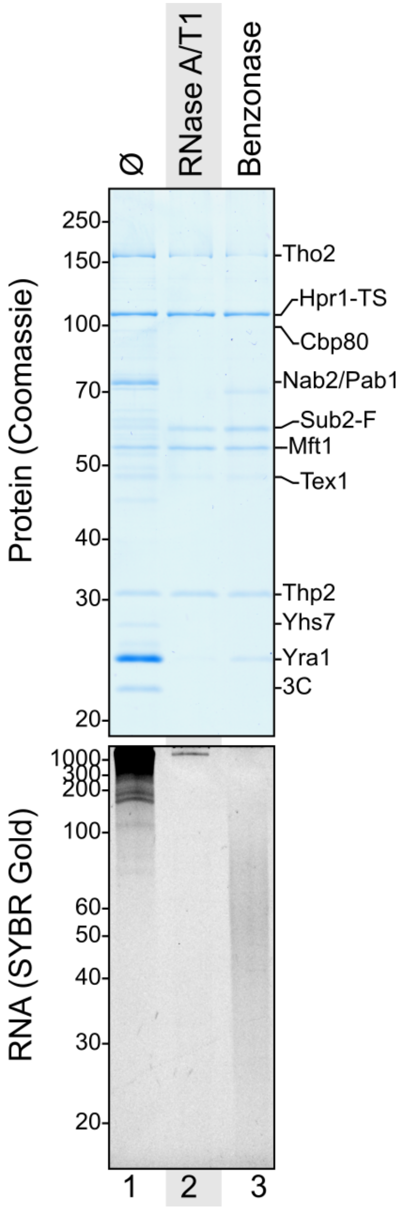

Supplemental Figure S3

RNase treatment analyses

Top: Coomassie-stained 10% SDS-PAGE gel of biotin eluates. Beads were treated with the indicated RNase prior to elution from the second affinity step.

Bottom: SYBR Gold stained 14% Urea-PAGE of Phenol-Chloroform-extracted RNA from an aliquot of the biotin eluates used in the above.
