# Supplementary material for: Nuclear mRNPs are compact particles packaged with a network of proteins promoting RNA–RNA interactions
Source: Genes Dev. 2023 Jun 1;37(11-12):505–17. doi: 10.1101/gad.350630.123 (PMC10393194; doi:10.1101/gad.350630.123)
Supplement: Supplemental Material [file supp_gad.350630.123_Supplemental_Fig_S4.pdf]

Supplemental Figure S4

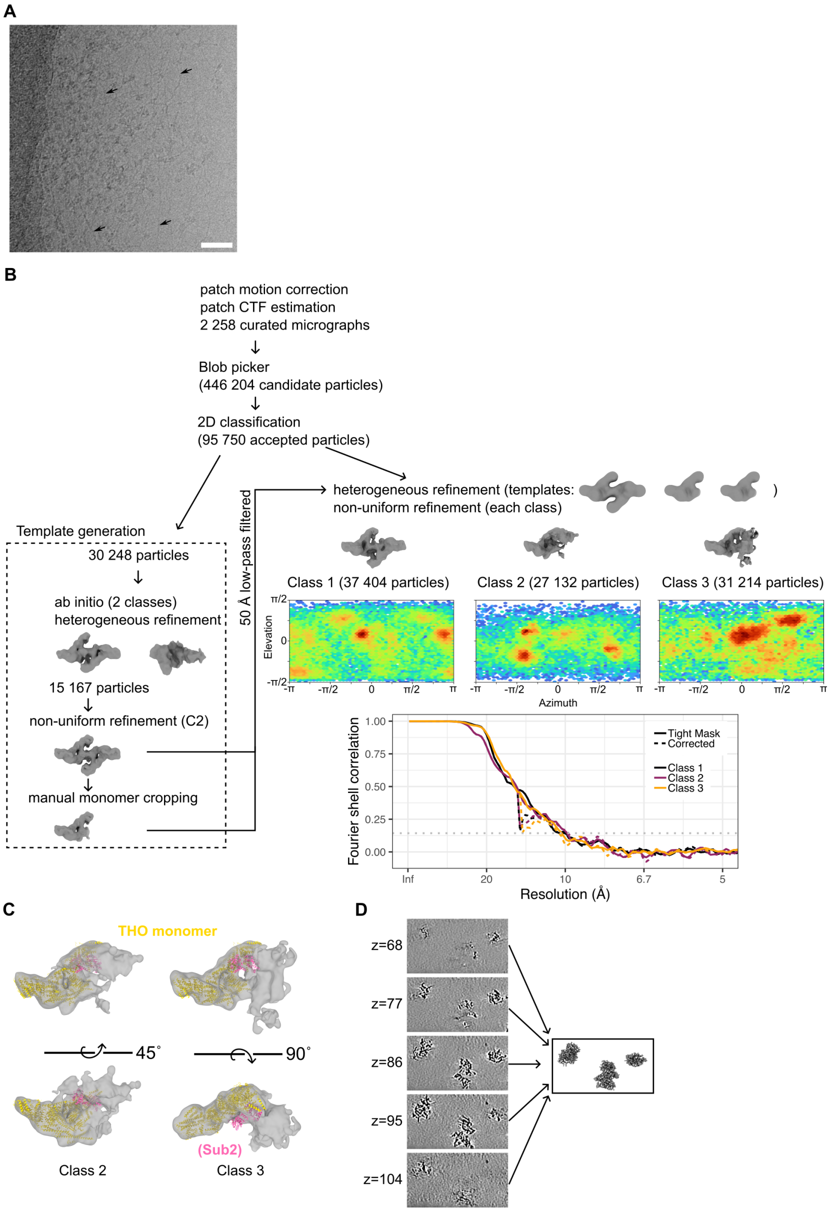

Electron microscopy analyses

A) Cryo-electron micrograph crop-out of particles eluted in native conditions in free ice, using a grid pre-coated with Epidermal growth factor (EGF) in an attempt to prevent adsorption on the carbon foil. The grid was pre-incubated 1 min with 4 µl EGF at 4 µM before blotting and sample application. Acquired at 2.5 µm defocus. Arrows point to fibers that presumably correspond to RNA from disassembled particles. Scale bar is 500 Å.

B) Processing scheme for single particle analysis of Benzonase-treated mRNPs with CryoSPARC. To allow for separation of particles showing clear dimers from particles where one THO monomer appears missing or in a variable position, a refinement with an imposed C2 symmetry was performed, and a manually cropped monomer was generated. 3D classification, including all accepted particles and using 50 Å low-passed filtered symmetric dimer and monomer as templates, yielded one class of a complete dimer (class 1). Heatmaps of particle orientations in each final reconstruction are shown, together with Fourier shell correlation plots of refined half-maps with mask auto-tightening before and after correction. "Gold standard" correlation level of 0.143 is indicated as a dotted gray line.

C) One monomer from a previously published structure of recombinant THO-Sub2 (pdb:7apx) (Schuller et al. 2020) was fitted into 3D reconstructions of the two least populated classes from B (gray volumes). THO is yellow, Sub2 is pink.

D) Five evenly spaced z-slices from the reconstructed and denoised tomogram (left) with the corresponding extracted volumes (right). Raw frames were corrected for beam-induced motion with MotionCor2 (Zheng et al. 2017) and further processed with IMOD (Mastronarde 1997).

For these analyses, micrographs were acquired with SerialEM (Schorb et al. 2019) using a beam-tilt based multi-shot acquisition scheme for faster imaging with a defocus target range spanning -1.5 to -3 µm. Each acquisition was fractionated into 40 frames with a total exposure of 59 e/Å^2 over 16 sec. Collected data were processed in CryoSPARC 4 (Punjani et al. 2017). Raw movie frames (2306) were aligned by patch motion correction, and per-micrograph contrast transfer function (CTF) was estimated with the patch CTF implementation. After removal of defocus outliers, 2258 micrographs were selected for

particle picking with the blob-picker using a range of 200 to 320 Å particle diameter. Adjusting template correlation and local power thresholds for the picks resulted in 446204 candidate particles extracted with a 324 pixel box size. Multiple rounds of reference-free 2D classification and removal of junk classes reduced this number to 95750 accepted particles.

The final three classes were further refined by non-uniform refinement (Punjani et al. 2020) and mask auto-tightening, giving three reconstructions reaching an approximate global resolution of 11 Å according to the Fourier shell correlation (FSC) cut-off criterion of 0.143 for the independent half maps (Rosenthal and Henderson 2003).

Mastronarde DN. 1997. Dual-axis tomography: an approach with alignment methods that preserve resolution. *Journal of structural biology* **120**: 343–352.

Punjani A, Rubinstein JL, Fleet DJ, Brubaker MA. 2017. cryoSPARC: algorithms for rapid unsupervised cryo-EM structure determination. *Nat Methods* **14**: 290–296.

Punjani A, Zhang H, Fleet DJ. 2020. Non-uniform refinement: adaptive regularization improves single-particle cryo-EM reconstruction. *Nat Methods* **17**: 1214–1221.

Rosenthal PB, Henderson R. 2003. Optimal determination of particle orientation, absolute hand, and contrast loss in single-particle electron cryomicroscopy. *Journal of molecular biology* **333**: 721–745.

Schorb M, Haberbosch I, Hagen WJH, Schwab Y, Mastronarde DN. 2019. Software tools for automated transmission electron microscopy. *Nat Methods* **16**: 471–477.

Zheng SQ, Palovcak E, Armache J-P, Verba KA, Cheng Y, Agard DA. 2017. MotionCor2: anisotropic correction of beam-induced motion for improved cryo-electron microscopy. *Nat Methods* **14**: 331–332.
