# Supplementary material for: Nuclear mRNPs are compact particles packaged with a network of proteins promoting RNA–RNA interactions
Source: Genes Dev. 2023 Jun 1;37(11-12):505–17. doi: 10.1101/gad.350630.123 (PMC10393194; doi:10.1101/gad.350630.123)
Supplement: Supplemental Material [file supp_gad.350630.123_Supplemental_Fig_S5.pdf]

Supplemental Figure S5

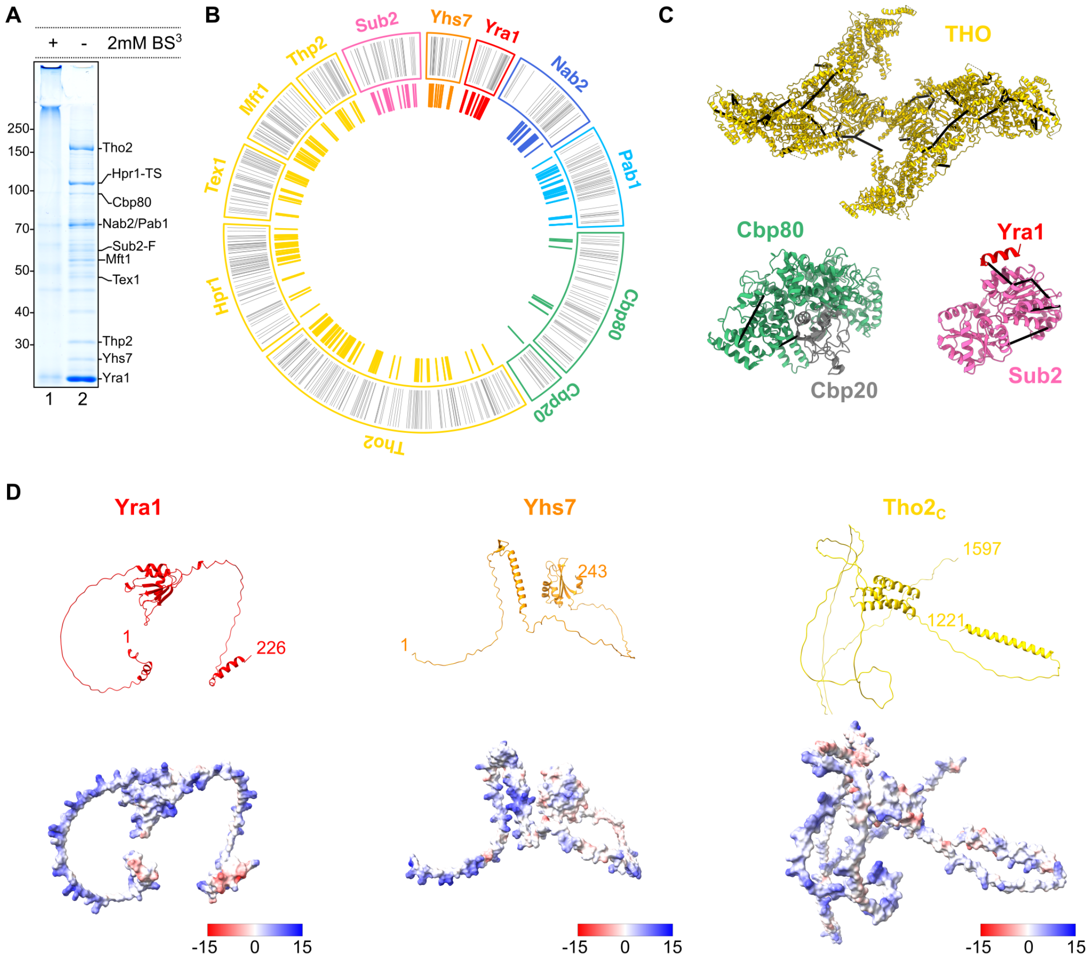

XL-MS analyses and AlphaFold predictions

A) Coomassie-stained 10% SDS-PAGE gels of TCA-precipitated native mRNP eluate with or without 2 mM BS^3 treatment for 20 minutes at 4˚C and subsequent quenching in 50 mM Tris.

B) Location of BS^3 mono-links in native particles as identified by mass spectrometry indicated as colored lines inside the circle. These originate from peptides where one half of the BS^3 cross-linker moiety reacted with Tris or with water. Gray lines in protein sectors indicate the position of lysines within each protein.

C) Experimental cross-links measured from purified mRNPs were mapped (black rods) on previously-determined structures of THO_{core} (Schuller et al. 2020), Sub2 (Ren et al. 2017) and Cbp80-Cbp20 (Li et al. 2019) using ChimeraX (Pettersen et al. 2021).

Li X, Liu S, Zhang L, Issaian A, Hill RC, Espinosa S, Shi S, Cui Y, Kappel K, Das R, et al. 2019. A unified mechanism for intron and exon definition and back-splicing. *Nature* **573**: 375–380.

Ren Y, Schmiege P, Blobel G. 2017. Structural and biochemical analyses of the DEAD-box ATPase Sub2 in association with THO or Yra1. *Elife* **6**.

Schuller SK, Schuller JM, Prabu JR, Baumgärtner M, Bonneau F, Basquin J, Conti E. 2020. Structural insights into the nucleic acid remodeling mechanisms of the yeast THO-Sub2 complex. *Elife* **9**.

D) AlphaFold structure predictions of indicated protein fragments (top) and their local electrostatic potential displayed with coulombic surface coloring in ChimeraX (Pettersen et al. 2021) (bottom). Scale unit is kcal/(mol·e) at 298 K.

For these analyses, peptides were separated at a flow rate of 300 nL/min by a gradient of buffer B (80% acetonitrile, 0.1% formic acid) from 5% to 30% over 40 min followed by an increase to 95% over 10 min then 95% over the next 5 min and finally the percentage of buffer B was maintained at 95% for another 5 min. The analytical column (30 cm, inner diameter: 75 microns; packed in-house with ReproSil-Pur C18-AQ 1.9-micron beads, Dr. Maisch GmbH) was heated to 60°C. The mass spectrometer was operated in data-dependent mode with survey scans from m/z 300 to 1650 Th (resolution of 60k at m/z = 200 Th), and up to 15 of the most abundant precursors were selected and fragmented using stepped HCD (with normalized collision energies of 19, 27, 35). The MS2 spectra were recorded with a

dynamic m/z range (resolution of 30k at m/z = 200 Th). Normalized AGC targets for MS1 and MS2 scans were set to 300 % and 100 %, respectively, within a maximum injection time of 25 ms for the MS1 scan. The maximum injection time was set to "auto" for the MS2 scans. Charge state 2 was excluded from fragmentation to enrich the fragmentation scans for cross-linked peptide precursors.

For the analysis, the raw data was processed using Proteome Discoverer (version 2.5.0.400) with XlinkX/PD nodes integrated (Klykov et al. 2018)). To identify the cross-linked peptide pairs, a database search was performed against a reduced FASTA containing the sequences of the proteins of interest and a contaminant database. BS^3/DSS was set as a cross-linker. Cysteine carbamidomethylation was set as fixed modification. Methionine oxidation and protein N-term acetylation were set as dynamic modifications.

Klykov O, Steigenberger B, Pektaş S, Fasci D, Heck AJR, Scheltema RA. 2018. Efficient and robust proteome-wide approaches for cross-linking mass spectrometry. *Nat Protoc* **13**: 2964–2990.
