# Supplementary material for: Nuclear mRNPs are compact particles packaged with a network of proteins promoting RNA–RNA interactions
Source: Genes Dev. 2023 Jun 1;37(11-12):505–17. doi: 10.1101/gad.350630.123 (PMC10393194; doi:10.1101/gad.350630.123)
Supplement: Supplemental Material [file supp_gad.350630.123_Supplemental_Fig_S6.pdf]

Supplemental Figure S6

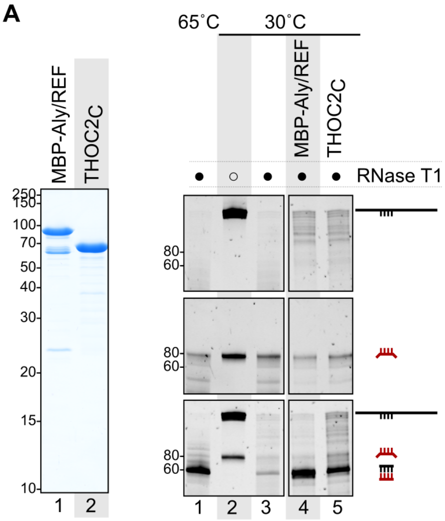

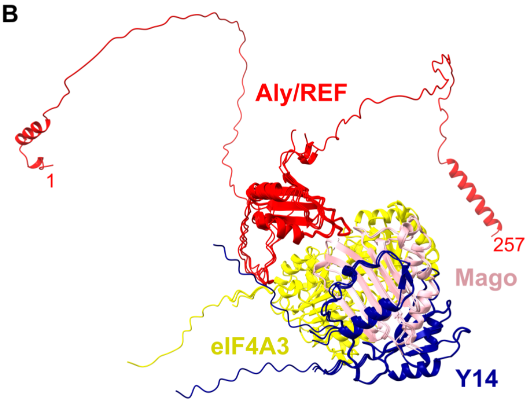

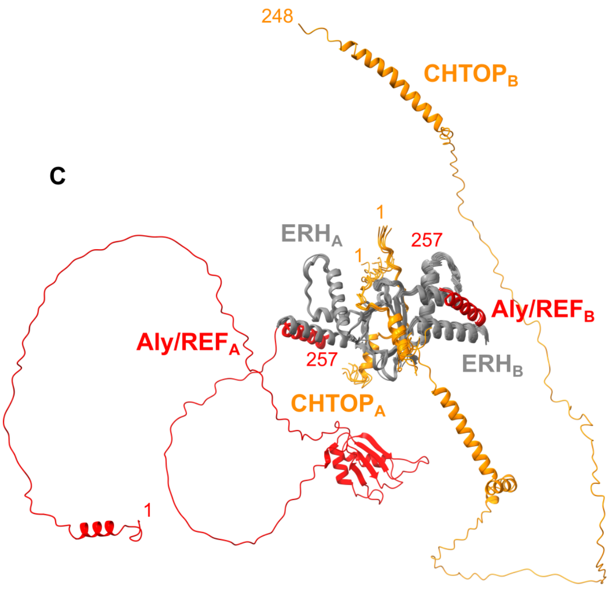

Extension of the IDR-network model to human nuclear mRNPs

A) On the right, RNA annealing assay as in Fig. 5D, using MBP-fused Human Aly/REF (full length, Yra1 orthologue) and the C-terminus of human THOC2 (residues 1280-1593, Tho2 orthologue). The assays were carried out as in Figure 5D: a template for probe transcription was obtained by annealing synthetic oligos (Sigma-Aldrich) for T7 promoter (AATTTAATACGACTCACTATAGG) and a 60 nt fragment with perfect complementarity to HHF1 flanked by 10 nt non-complementary overhangs on either side (GTGTCTTTCTTTCTAAGAGATAACATCCAAGGTATTACTAAGCCAGCTATCAGAAG ATTAGCTAGAAGAGCACCACACCCTATAGTGAGTCGTATTAAATT).

The quality of the purified recombinant proteins used in the assay are shown in the Coomassie-stained SDS gel on the left. THOC2 (1280-1593) was cloned in a bacterial expression vector fused at its 5'-end to a sequence coding for a His-SUMO-tag cleavable with SENP2 protease, and was purified similarly to was described in materials and methods of the yeast Tho2C (1221-1597). Full length MBP-Aly/REF was cloned in a bacterial expression vector fused at its 5'-end to a sequence coding for a His-MBP cleavable with 3C protease. MBP-Aly/REF was purified using Nickel-based affinity chromatography followed by an additional purification step to remove remaining nucleic acids: after a 15-fold dilution in buffer A (20 mM Tris pH 8.0, 100 mM NaCl, 2 mM DDT), the proteins were bound to a heparin chromatography column (Cytiva) and eluted within a 20 CV gradient of buffer B (20 mM Tris pH 8.0, 1 M NaCl, 2 mM DDT) and dialyzed against a storage buffer (Hepes 20 mM pH 7.5, 350 mM NaCl and 2 mM DDT). The bands in the Coomassie-stained gel were submitted to mass-spec analysis, which identified them as corresponding to the full-length and proteolytic fragments of MBP-Aly/REF.

B) AlphaFold Multimer predictions of full-length human eIF4A3, Mago, Y14, Aly/REF complex. Four predictions converged on the same model for the structured core of the EJC, comprising Mago, the N-terminal and RRM domains of Y14 (which wrap around Mago, as observed in Fribourg et al. 2003), the helicase core of eIF4A3 (which interacts with Mago-Y14 as previously observed in the structures of the EJC core, Bono et al. 2006, Andersen et al. 2006); the Aly/REF RRM domain (at a loop residues 140-143) and an N-terminal motif (residues 82-92, in agreement with biochemical data in Gromadka et al., 2016). We note that

the AlphaFold predictions were carried out prior to the release of the PDB 7ZNJ (Pacheco-Fiallos et al., 2023) and thus did not incorporate the information from the experimental model in the predictions. Remarkably, the interacting regions in the AlphaFold predictions align with those in the cryo-EM reconstruction model of EJC truncation mutant, except that the interactions occurred in a monomeric form of the EJC. In addition to the structured core (shown with independent models superposed to each other), EJC subunits have IDRs that extend from the core (shown for a single model). The IDRs are particularly prominent for Aly/REF.

Andersen CBF, Ballut L, Johansen JS, Chamieh H, Nielsen KH, Oliveira CLP, Pedersen JS, Seraphin B, Le Hir H, Andersen GR. 2006. Structure of the exon junction core complex with a trapped DEAD-box ATPase bound to RNA. *Science* **313**: 1968–1972.

Bono F, Ebert J, Lorentzen E, Conti E. 2006. The crystal structure of the exon junction complex reveals how it maintains a stable grip on mRNA. *Cell* **126**: 713–725.

Fribourg S, Gatfield D, Izaurralde E, Conti E. 2003. A novel mode of RBD-protein recognition in the Y14-Mago complex. *Nat Struct Biol* **10**: 433–439.

Gromadzka AM, Steckelberg A-L, Singh KK, Hofmann K, Gehring NH. 2016. A short conserved motif in ALYREF directs cap- and EJC-dependent assembly of export complexes on spliced mRNAs. *Nucleic Acids Res* **44**: 2348–2361.

C) AlphaFold Multimer predictions of full-length human ERH with CHTOP or Aly/REF were carried out based on cross-linking mass spectrometry information of native human nuclear mRNPs (Figure 3 in Pacheco-Fiallos et al., 2023). The predictions incorporate known structural information that ERH is a homo-dimer (Hazra et al., 2020, Perez-Borrajero et al. 2021). Multiple predictions converged on the same structural core formed by the ERH homodimer, the C-box helix of Aly/REF (residues 241-256, 17 models) and the N-terminus of CHTOP (residues 1-28; 14 models). Note that two Aly/REF C-box and two CHTOP N-terminus elements can potentially bind at the same time on the ERH homodimer (see A and B labels for each protomer).In addition to the structured core (shown with independent models superposed to each other), Aly/REF and CHTOP have IDRs that extend from the core (shown for a single model and for a single protomer for clarity). Note the Aly/REF C-box would be accessible when Aly/REF is either alone or when associated with eIF4A3-Mago-Y14 (panel B), but not when Aly/REF is bound to UAP56 (Ren et al., 2017).

Hazra D, Andrić V, Palancade B, Rougemaille M, Graille M. 2020. Formation of S. pombe Erh1 homodimer mediates gametogenic gene silencing and meiosis progression. *Sci Rep* **10**: 1034–11.

Perez-Borrajero C, Podvalnaya N, Holleis K, Lichtenberger R, Karaulanov E, Simon B, Basquin J, Hennig J, Ketting RF, Falk S. 2021. Structural basis of PETISCO complex assembly during piRNA biogenesis in C. elegans. *Genes Dev* **35**: 1304–1323.
